# The hemophilia quality of life scale: a systematic review

**DOI:** 10.3389/fpubh.2024.1294188

**Published:** 2024-02-06

**Authors:** Xin Wang, Zhixiao Li, Lele Li

**Affiliations:** ^1^School of Maritime Economics and Management, Dalian Maritime University, Dalian, China; ^2^School of Labor and Human Resources, Renmin University of China, Beijing, China

**Keywords:** quality of life, hemophilia, quality of life scale, well-being, life

## Abstract

**Purpose:**

Quality of life refers to the degree of well-being a person feels. The development of a hemophilia-targeting quality of life scale is important for hemophiliacs and their treating physicians.

**Methods:**

Textual analysis. First, a review of studies on quality of life, hemophilia and related quality of life scales was conducted; Subsequently, two rounds of systematic searches of the Springer database were conducted to filter the literature on universal quality of life scale studies and hemophilia-targeting quality of life scale studies by title and abstract, and then textual analysis was performed.

**Results:**

The former included 77,456 articles, 26,117 chapters and 7,086 related bibliographies, while the latter initially retrieved 211 entries articles, 118 chapters and 43 related bibliographies. Through filtering, the former contains 22 documents, yielding 1,431 valid word stems, and the latter contains 9 documents, yielding 1,541 valid word stems.

**Conclusion:**

While universal quality of life scales mostly fit into the broad framework of WHOQOL- BREF, the development of hemophilia-targeting quality of life scales inclines towards pains that patients suffer and technology advances in pharmaceutical. The current hemophilia QOL scales are mainly based on the HR-QoL, others mainly based on the HR-QoL as the master version. At the same time, the popularization of existing hemophilia quality of life scales in developing countries like China is not high, and the development of hemophilia quality of life scales is insufficient.

## Introduction

According to the definition of the World Health Organization, quality of life is the experience of individuals in different cultures and value systems with their goals, expectations, standards, concerns and related living situations (1). At present, quality of life has gained significant attention from international academic research. Following the change of modern medical model, it is widely used in the screening of clinical treatment methods for chronic diseases, the evaluation of the effect of preventive interventions and the allocation of health resources and other aspects (2). The selection of the measurement dimension of quality of life greatly depends on the understanding of quality of life, and this choice takes the scale as the main presentation form in the transformation of academic achievements.

Health is considered to be an important indicator of quality of life. In a narrow sense, health refers to being healthy at the physical level. Simply put, a person is considered healthy if he is in a state of non-illness. However, it is clear that an individual’s physical health is incomplete to illustrate the entire state of health. Along with cross-disciplinary and interdisciplinary knowledge, on an epistemological level, health should include psychological feelings, especially what is called “the subjective well-being.” It is worth mentioning that in the United States around 20c50s, the measurement of subjective well-being has always been in a pivotal position. In the late 18th century, the British utilitarian ethicist Bentham measured happiness from the calculation of pleasure and pain experienced by people (3). Siddwick (4) challenged this and argued that the definition derived from the assumption of self-interested hedonism was vague. Neugarten et al. published the ISL scale in 1961, which involved many self-concepts, one of which is an important dimension of the psychological quality of happiness and optimism (5). At present, no significant distinction has been identified between happiness and mental health, and the two can even be interchangeable. After all, “happiness,” as a subjective feeling, is the self-evidence of mental health. Another point is that there are external factors that affect happiness, and this also suits a mentally healthy person. On the other hand, an individual without happiness is more likely to induce psychological problems, which has been confirmed by most literature. From an individual perspective, high happiness equals good mental health.

Of course, a person’s quality of life is not all about health. Whether physical or mental health, it is limited to the range of “individual.” The assessment of the quality of life should also include the surrounding environment. Judging from the development of sociology, no matter how much previous sociologists disagree on the behavior pattern of human beings on a small scale, the agreement they have reached is that human beings are confined by society. This has nothing to do with whether humans exist *a priori* or *a posteriori* in society. What counts is that the social environment, the manifestation of the various relationships between people, can greatly affect an individual’s experience of life. The most typical example is the theory of Social Exclusion (6), which argues that social exclusion is a breakdown of the social bond between individuals and society, and those isolated individuals suffer from economic, political and cultural exclusion, which makes life harder. Excluded social groups struggle to gain resources to improve their quality of life, resulting in a negative feedback loop. In addition, the physical environment, is also an important external factor affecting an individual’s quality of life. For example, clean water, clean air, convenient transportation and so on will improve people’s quality of life. On the one hand, providing good environment can improve an individual’s physical health. The important evidence of infectious diseases (7) is that people living in poor environment and dirty districts are more easily to get infected.

To sum up, the quality of life can be divided into the following structure, which is mainly composed of internal physical and mental health and the surrounding environment including the natural (or physical) and social environment. This conclusion has affinity with the World Health Organization’s understanding of quality of life as “a state of physical, mental, and social well-being, rather than merely the absence of disease and infirmity.”

Aaronson et al. (8) also agree that quality of life is a multi-dimensional concept, involving many aspects of physical functioning, psychological and social satisfaction. On this basis, Meyerrowitz et al. also proposed that the measurement of life quality must include subjective indicators (2). Chonghua (9) summarized the measurement methods of life quality into five methods: interview method, observation method, subjective report method, symptom pattern inspection method and standardized scale. Some scholars believe that the standardized scale method is more comprehensive at present.

At present, there are many types of universal scale, that is, generic core. According to scholar Chonghua (9) summary in 2000, the generic quality of life scale can be summarized in Table 1.

**Table 1:** Basic elements of the QoL.

What QoL should contain		
Physical health	←	Physical environment
↑↓		
Psychological health	←	Social environment

It is not difficult to find that there are universal and specific quality of life scales. The universal scale, such as GHO, NHP, IMH, WHO-QoL, focuses on the whole human group, while the special scale is generally developed for different groups on the basis of the universal scale. For example, ADL is aimed at the older adult group, LASA is to measure the quality of life of breast cancer patients, and LIC scale is to measure the life function index of cancer patients. However, it is worth noting that the universal scale and the specific scale are not completely separate, and their dimensional measurement division cannot be separated from the framework discussed above. For example, the Functional Assessment OF Cancer Therapy developed by Bonomi and Cella (10), Chicago Medical Center, United States, is a scale group composed of common modules and specific cancer subscales. The common module includes 34 items, which are composed of physical status, social and family environment, relationship with doctors, emotional status and functional status. Quality-of-life measurements for specific cancers are composed of both generic and specific modules. Undeniably, the universal scale represents most of the dimensions of quality of life and is also the basis for the further development of the specific scale. The development of specific scales for patients with various diseases is an important direction for the facilitation of the social quality scale. This study focuses on the development and use of the hemophilia specific scale (Tables 2–4).

**Table 2:** Scales of QoL.

Scales	Source	Type
KPS (Karnofsky Performance Status)	Karnofsky (11)	Specific (targeted for patients with cancer)
ADL (Index of Independence in Acitivity of Daily life)	Katz et al. (12)	Specific (targeted for the older adult)
GHO (General Health Questionnaire)	Berwick et al. (13)	General
NHP (Nottingham Health Profile)	Hunt et al. (14)	General
IMH (Index for Measuring Health)	Grogono and Woodgate (15)	General
SIP (Sickness Impact Profile)	Bergner et al. (16)	General
LASA (Linear Analog Self-Assessment, LASA)	Prestman and Baum (17)	Specific (targeted for patients with breast cancer)
QWB (Quality of Well Being Index)	Kaplan et al. (18)	General
QL-Index (QL-Index)	Spitzer et al. (19)	General
FLIC (The Functional Living Index-Cancer)	Schipper et al. (20)	Specific (targeted for patients with cancer)
MOS SF-36 (Medical Outcome Study)	Stewart and Ware (21)	General
CARES (Cancer Rehabilitation Evaluation System)	Schag and Heinrich (22)	Specific (targeted for patients with cancer)
EORTC QLO-C30	Aaronson et al. (8)	Specific (targeted for patients with cancer)
WHOQOL-100	WHO (23)	General
FACT	Cella	Specific (targeted for patients with cancer)

**Table 3:** WHOQoL related domains.

WHOQoL		Corresponding coding
1	PHYS	PHYS
	Pain	Pain
	Energy	Energi
	Sleep	Sleep
2	Psychological domain	PSYCH
	Positive feelings	Pfeel
	Thinking ability	Think
	Esteem	Esteem
	Body	Body
	Negative emotions	Neg
3	Independence	IND
	Mobility	Mobil
	Activity	Activ
	Reliance on medic	Medic
	Working ability	Work
4	Social domain	SOCIL
	Personal relationship	Relat
	Supports from society	Supp
	Sex	Sexx
5	Environmental domain	ENVIR
	Social safety	Safety
	Home	Home
	Financial conditions	Finan
	Medical and social service	Service
	Information to learn	inform
	Chances for leisure	Leisur
	Environment	Envir
	Transportation	Transp
6	Spiritual domains	DOM6
	Spiritual domains	Spirit

**Table 4:** Keywords for searching.

Screening of research literature on universal quality-of-life scale	Screening of the research literature on the hemophilia quality of life scale
With all of the words	With all of the words
Measurement of quality of life	Measurement of quality of life
With the exact phrase	With the exact phrase
Quality of life	Quality of life
With at least one of the words	With at least one of the words
Quality of life	Hemophilia
Final outcome:*n* = 22	Final outcome:*n* = 9

To develop the disease—related quality of life scale, understanding the corresponding disease is a prerequisite. Hemophilia is an inherited hemorrhagic disorder caused by the deficiency in clotting factors that can cause joint damage and even disability (24). There are three classifications of hemophilia, including Hemophilia A and Hemophilia B, which are inherited in an X-linked manner, while Hemophilia C is inherited in an autosomal recessive mode. Hemophilia C is rare. Hemophilia is more common in men, and women are usually carriers (25). As treatments have improved, the overall situation for hemophiliacs has improved. But scholars like Shen believe that even so, because of lifelong medication and joint damage, hemophiliacs suffer from chronic stress, which challenges their quality of life. Through the evaluation of the anxiety of hemophiliacs, it is found that the quality of life of hemophiliacs is affected by three aspects. First of all, financial condition is an important consideration for hemophiliacs. The poorer the financial condition, the less likely they are to be in delight. This is related to the fact that hemophilia requires lifelong treatment. Without financial support for treatment, patients can easily become anxious. Then there is the burden of pain. Hemophilia is characterized by persistent bleeding, typically from the joints. Patients with more frequent bleeding are more likely to experience anxiety. At the same time, family factors are also influencing factors for depression in hemophiliacs. On the one hand, families in less developed areas are more likely to panic due to the deficient understanding of hemophilia, aggravating the psychological burden; On the other hand, hemophilia can bring negative effects to patients and their families at different levels. Studies prove that hemophiliacs face a great psychological burden.

At present, there are generally two ways to evaluate the quality of life of hemophiliacs. One is the method adopted by Yaoguang and Ling (26). Based on a universal scale such as SF-36 or WHO-QOL, and combined with relevant demographic and hemophilia data in terms of specificity, information on gender, age, occupation, bleeding frequency, bleeding site and joint involvement was obtained. At the same time, self-rating anxiety scale (SAS) was introduced and self-rating depression scale (SDS) was used for specificity analysis. The WHO-QOL Scale is divided into six dimensions, physical, psychological, independence, social relations, environment and beliefs. The specific analysis is as follows:

It is noteworthy that the hemophilia-targeting scales are not the same as HR-QoL. Health realated QoL is generally considered to reflect the impact of disease and treatment on disability and daily functioning (27). The impact of disease is one side that affect pacients’ quality of life. Other societal factors as are indicated in the universal QoLs can still influence patients’ life. Thus hemophilia-targeting scales impose comprehensive consideration on patients.

However, the development of hemophilia-targeting scales is still inadequate, and the existing hemophilia-targeting scales are miscellaneous. The evolution of hemophilia scales has been scarcely studied, and thus the development of hemophilia scales is unclear. It is complicated to recognize the differences between hemophilia quality of life scales and other quality of life scales or even rare disease quality of life scales. In addition, the existing hemophilia quality of life scales is deficient in systematic quality checks, especially in terms of cross-sectional comparisons of scale content, whether they fit into the multiple dimensions of life assessment of hemophiliacs, and whether they can correctly assess the quality of life of hemophiliacs. Moreover, the existing measuring tools are mostly focused on European and American countries, and the popularization of existing quality of life scales in developing countries like China is not yet evident, considering the applicability of the scales and differences among regions. Therefore, three questions are addressed respectively: How do universal quality of life scales differ from hemophilia-targeting quality of life scales? What are the priorities and shortcomings of the existing versions of the scales? How is it being used in developing countries like China? Hence, in line with the approach of identifying problems to address them, these three issues play a crucial role in advancing the overall research. Given that the Quality of Life scale and its utilization report are predominantly presented in textual format, this paper initiates by conducting text analysis to comprehend the context surrounding these three problems. The subsequent section outlines the specific research methods employed in this study.

## Method

### Search strategies

Using the Springer database as the main database, the author filtered the literature related to quality of life scales by keyword search, and eliminated invalid documents and specific operational documents, and selected the review literature on quality of life scales by title and abstract. The search yielded 77,456 articles, 26,117 chapters, and 7,086 related titles, and the result was a compilation of 22 related literature. In the study of hemophilia-targeting scales, the authors searched by keyword. A total of 211 articles, 118 chapters and 43 related books were screened for relevant literature, and after excluding irrelevant literature, a total of 9 hemophilia quality-of-life scale development papers were selected.

When it comes to selecting article on universal quality of life, the author initially chose to search “measurement of quality of life.” However, since QoL was the basic element in this study, restrictions were set that outcomes should at least involve “quality of life.” Then, the author chose “article” as the content type in Springer. In order to meet the quality of random sampling, 50 articles were chosen randomly in the database. Then the author paid attention to abstracts to involve associated articles and 22 of them met the criteria that, first, the literature should concern about the development of QoLs, and second, it should inspect the quality of tools. Broadly the same searching strategy was used to compile sources on Hemophilia Quality of Life Scale, while “hemophilia” must be included and the literature should concern about the development of Hemophilia Quality of Life Scales. 9 articles were finally chosen.

### Textual analysis

Considering the linguistic composition of English characters and other issues, the Porter stemming method is used for encoding, a stem extraction based on suffix stripping (28),when using the software. A total of 1,431 valid word stems were extracted and the frequency counts were calculated. The four dimensions of the WHOQOL-BREF questionnaire were then used to filter out: “physician” “pain” “energi” “psycholog” “psychiatri” “feel” “perceiv” “bodi” “neg” “independ” “mobil” “activ” “work” “social” “relat” “support” “environment” “safeti” “home” “financi” “servic” “inform” “transport” “domain” “spiritu.” The Porter stemming method was also used to extract 9 documents on quality of life in hemophilia, and a total of 1,541 groups of high-frequency stems were screened.

It is noteworthy that in the 22 literature, the author excluded titles, abstracts and references and remained the rest of the literature encoded. This approach is based on the following considerations: First, titles, abstracts, and references are a re-distillation of the content of the literature and lead to unnecessary duplication of data. Excluding them can effectively reduce the burden of data in text analysis. Second, the content of other parts, including introduction, literature review, data analysis and conclusion, can effectively show the frequency of keyword analysis. Third, the scale design will be based on the development of previously verified scales. The text of the article, especially the literature review, can reveal the author’s reference credentials. In order to investigate the evolution of the scale and test whether it fits the module of WHO-QoL, it is necessary to conduct frequency check on the full text.

## Results

Most of the scales commonly used in studies on quality of life refer to the WHOQOL-BREF template. An important reason for this judgment is the absence of extremes in the frequencies of the four word-groups, which show a smooth distribution pattern. In addition, the cross-sectional comparison of frequencies reveals that the psychological factors and social relations domains are given significant attention in the quality of life scales. Among the psychological domains, individuals’ thinking, learning ability, memory and attention (“perceiv”) and body conditions along with appearances (“bodi”) are weighted higher. In the social relations domain, personal relationships (“relat”) and satisfaction with required social support (“supp”) are balanced and play an equally important role in the assessment of the quality of life of individuals. It is noteworthy that in conducting the original-stem extraction, sleep (“sleep”), self-esteem (“esteem”), sexual life (“sexx”), opportunities for and participation in leisure and recreational activities (“leisur”), and dependence on medication and medical treatments (“medic”), all of which are part of WHOQOL-BREF measurement, have not been found in the 22 articles. One possible reason for this is that these six aspects have not been studied in the 22 papers. The other is that the stemming process automatically eliminated stems with a frequency of less than 2. In any case, these six aspects have not received sufficient attention and discussion during the evolution of the QOL. Besides, “haemo-” and hemophilia-related stems were not retrieved among the 1,431 sets of stems, indicating that the hemophilia-targeting QoL was not developed as a typical type of scale.

The same WHOQOL-BREF dimensional classification and word categorization were used to obtain the Table 5. The frequency counts show that, unlike the general quality of life scale, pain is given a high weight in the assessment of quality of life in hemophilia. Physiological pain perception is currently an important evaluation indicator in the measurement of quality of life in hemophilia. In addition, the low frequency of “psycholg” shows the deficiency of development and measurement of psychological dimensions in the hemophilia-targeting quality of life scale. Notably, the high frequency of dependence on medication and medical treatment (“medic”) relates to the fact that hemophilia is a disease, and that medical advances and optimization of treatments play an elemental role in the hemophilia quality of life measurement. From another point of view, medical advances can effectively alleviate physical pain, which also increases frequency of “medic.” The same as the universal quality of life scale, the Hemophilia Scale is deficient in the development and measurement of energy(“energy”), sleep(“sleep”), esteem(“esteem”), sex life(“sexx”), and participation in leisure (leisur) (Tables 6, 7).

**Table 5:** Outcomes of textual analysis on universal QoLs.

Stem	Exact words	Part of speech	Frequency	Line number
life		noun.	325	207
Qualiti	Quality:305, qualities:1	noun.	306	206
Item	Items:83	noun.	209	83
Particip	Participants:136, participating:16, participation:13, participant:11, participated:10, participate:6, participative:2	noun.	194	126
Citi	Cities:105, city:84	noun.	189	89
Measur	Measurement:83, measures:34, measure:18,measuring:16, measurements:12, measured:4, measurable:1	noun.	168	130
Chines	Chinese:130	noun.	130	85
Health		noun.	94	67
Model	Models:23, modeling:1	noun.	87	51
Assess	Assessment:37, assessments:19, assessing:12, assessed:11	verb.	85	71
WHOQoL-		noun.	77	61
Diseas	Disease:42, diseases:7	noun.	49	36
Physician	Physicians:5	plur.	9	8
Pain		noun.	7	7
Energi	Energy:10	noun.	10	9
Psycholog	Psychological:42, psychology:3	adj.	45	43
Psychiatri	Psychiatry:7	noun.	7	7
Feel	Feelings:4, feeling:2, feels:1	noun.	12	12
Perceiv	Perceived:33, perceive:11, perceives:3	verb.	47	42
Bodi	Body:24	noun.	24	15
Neg	Negative:6, negatively:2	adj.	8	6
Independ	Independent:6, independently:1, independence:1	adj.	8	6
Mobil	Mobility:4, mobile:2, mobilate:1, mobilizes:1	noun.	8	8
Activ	Activities:8, activity:5, active:2	plur.	15	13
Work	Working:1	noun.	18	11
Medic		adj.	33	28
Social	Socially:1	adj.	66	46
Relat	Related:17, relations:3, relate:3, relation:2, relates:1	verb.	26	24
Support	Supported:2, supports:2, supportive:1	verb.	23	18
Environment	Environmental:11	adj.	11	10
Safeti	Safety:11	noun.	11	10
Home	Homes:2	noun.	12	9
Financi	Financial:13, financially:2	adj.	15	13
Servic	Services:8, service:2	noun.	10	8
Inform	Information:16, informs:2, informative:2	noun.	20	16
Transport	Transportation:1	noun.	16	10
Domain	Domains:15	noun.	49	26
Spiritu	Spirituality:5, spiritual:3	noun.	8	8

**Table 6:** Outcomes of textual analysis on existing Hemophilia QoLs.

Stem	Word	Part of speech	Frequency	Line number
Hemophilia		noun.	578	332
Patient	Patients:305	plur.	366	235
Life		noun.	312	242
Health		noun.	259	208
Qualiti	Quality:247	noun.	247	201
SEVER	Severe:140, severity:59, several:17, severely:2	adj.	218	144
Treatment	Treatments:10	noun.	179	124
Diseas	Disease:102, diseases:72	plur.	174	139
Physic	Physical:93	adj.	93	71
Pain		noun.	98	67
Psycholog	Psychological:5, psychology:3	noun.	8	8
Perceiv	Perceived:58, perceive:1	verb.	59	43
Bodi	Body:29, bodies:1	noun.	30	26
Neg	Negatively:13, negative:8	adj.	21	17
Mobil	Mobility:9	noun.	9	9
Activ	Activity:45, activities:16, activated:2, Active:2	noun.	65	55
Medic	Medical:66, medication:7, medications:2	adj.	75	50
Work	Worked:2, working:2, works:2	noun.	34	26
Social	Socializing:1	adj.	63	56
Relat	Related:44, relation:10, relations:1	verb.	55	51
Support	Supported:3, supports:1	noun.	25	25
Environment	Environmental:10	adj.	10	8
Psychometr	Psychometric:11	noun.	11	10
Finan	Financial:12	adj.	12	11
Servic	Services:8, service:2	plur.	10	9
Inform	Information:27, informal:11, informed:7	noun.	45	41
Leisur	Leisure:3	noun.	3	3
Transport	Transportation:2	noun.	11	5
Domain	Domains:23	noun.	33	25

**Table 7:** Extractions from “age.”

Stem	Word	Part of speech	Frequency	Line number
Ag	Age: 119, aged:28, ages: 4	verb./noun.	151	102
Year	years:106	plur.	126	87
Adult	adults:55	plur.	103	91
Children		plur.	183	142

Besides it should be noted that age had an important grouping role in the development of the Hemophilia-targeting Scale. WHOQOL-BREF and other quality of life scales do not intentionally distinguish between age levels, but during stemming, researchers found that the “adult” and “children” holds high frequency. Hemophilia in adults differs from hemophilia in children. Child-targeting tools should be developed according to children’s developmental status and relevant aspects of different age groups. For children, parental reports of child well-being are necessary, whereas for teenagers, comparisons between child and parental perspectives are inherently interesting.

**Figure 1:**
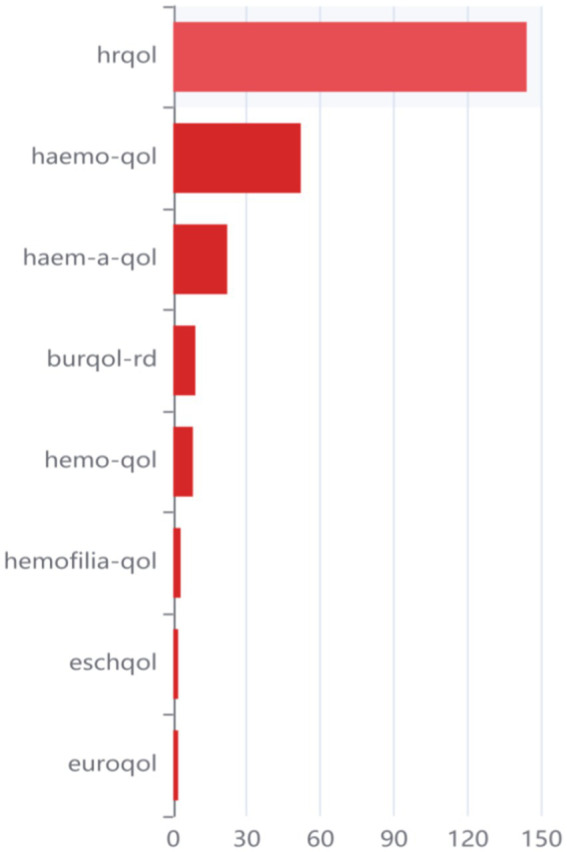
Frequency ranking in QoLs.

In Figure 1, the word frequency ranking related to Quality of Life (QoL) is presented. Considering the two different spellings, “haemo” and “hemo” used in reference to hemophilia, both variations are included in the statistical analysis. Among six hemophilia-targeting scales developed so far, the HR-QoL was widely used among the other developed hemophilia scales in the literature selected by researchers, implying that Health-Related Quality of Life holds greater significance and merits further attachment.

**Figure 2:**
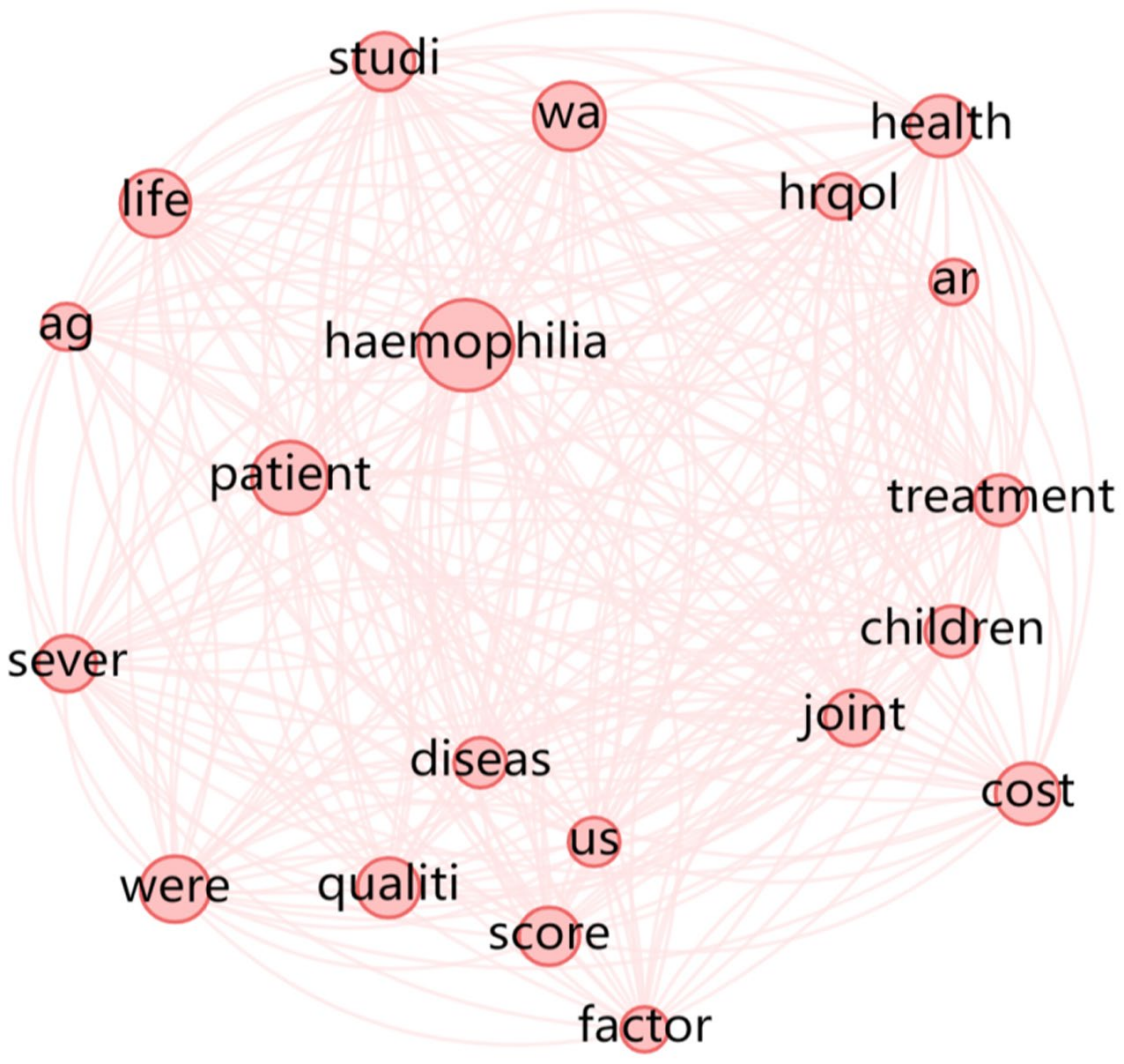
Stem relationship diagram of the hemophilia-specific quality of life scale.

**Figure 3:**
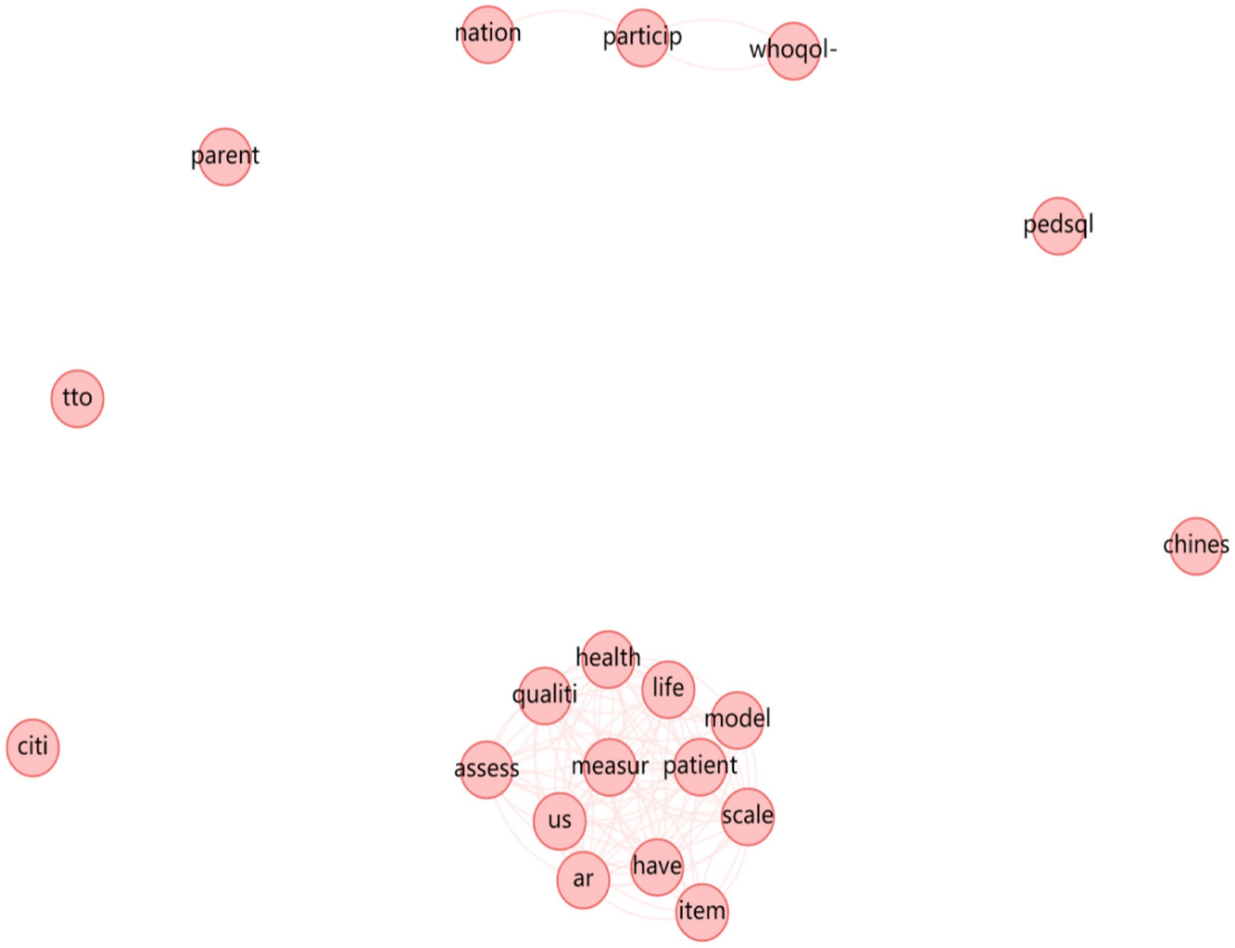
Stem relationship diagram of the universal quality of life scale.

Figures 2, 3 illustrate the strength of associations among different word stems, based on Hemophilia-Specific Quality of Life Scale and Universal Quality of Life Scale. The size of each circle represents the frequency of occurrence, with larger circles indicating higher frequencies. The smaller circles represent components with lower influence. The connections between circles reflect the strength of associations, with a higher number of connections indicating a closer relationship between the corresponding elements, which can be reflected by undirected edges between two word stems. In other words, if there are more edges between two word stems, it means that the association when it comes to compiling relevant scales between these two word groups is stronger and tighter. It can be further concluded from the stem relationship mapping that the HR-QoL scale is strongly associated with children and is largely the master frame of other hemophilia scales, which means other types of hemophilia scales are largely based on the HR-QoL.

Finally, it is worth noting that during the relationship-mapping of word stems for the quality of life scale, although the frequency of Chinese was 130 in the word frequency statistics, it was isolated form “health” and “quality” in the relationship map, and therefore a substantial association did not exist. The use and testing of the universal quality of life scale in China is still open to question. In addition, the frequency count of “Chinese” in the literature of hemophilia quality of life scales is only 6, which shows that there is a lack of development of hemophilia-specific quality of life scales in China. Thus, the utilization rate of existing scales, including the HR-QoL, is even low in mainland China. To develop particular scales for corresponding regions, also catering for both children and adults, is of great significance.

## Discussion

In the analysis of development papers related to the universal QOL scale, this paper first assumes that most development tools are based on WHO-QoL dimension evaluation, and namely, further QoL development tools are evolved from WHO-QoL. WHO-QoL is therefore used as the main coding framework and text analysis within this framework. This hypothesis arises because previous literature mentions the WHO-QoL as the most widely used quality of life scale (29). As is illustrated in the introduction, the scale also fits into the framework of the quality of life evaluation. It is expected to see from the word frequency statistics the degree of further development on this basis, especially the degree of further exploration of each dimension of quality of life.

At the same time, it is worth noting that the hemophilia-targeting QoL scale is a comprehensive evaluation of the patients (30), and is not only limited to the disease itself. Thus unlike the HRQoL alone, the second part of this study aims to observe to what extent the existing hemophilia QOL scale fits the framework of the universal QOL scale. Through the comparison of frequency gap, it can be seen that the further development of hemophilia quality of life scale also requires efforts in patients’ energy, sleep, sex and leisure and entertainment. Moreover, because of the large gap between adults and children, the study suggests the development of hemophilia quality of life scales suitable for adults and children. Furthermore, conducting relevant research on the audience of the targeted scale, such as using grounded theory to interview patients, doctors, and healthcare institutions regarding their actual experiences with its usage, can provide further assistance in amelioration.

The development of a hemophilia-specific scale is a lengthy process, especially in developing countries. Firstly, it is important to refer to existing scales, including their principles, basic structures and the classification of pain levels, while striving to encompass various aspects that impact the quality of life of hemophiliacs. Secondly, it is necessary to clarify the differences among distinguished groups, such as adults and children as was suggested above. Thirdly, in terms of specific measurement methods, local statistical practices should be followed, such as frequency of medical statistics, units of measurement, and alignment with the healthcare system. Fourthly, it is crucial to establish a comprehensive feedback mechanism to continuously observe the pain characteristics and different life issues caused by hemophilia in patients during the scale development process, and make adjustments accordingly.

Generally, this study focused on the Springer database and conducted a preliminary screening of relevant literature in the Springer database based on abstracts and titles. The selected databases are limited, which may affect the output of the results. Secondly, the Springer database is dominated by English-language literature, with insufficient coverage of localized studies from other language countries, which may lead to bias in the assessment of the practical use of the hemophilia quality of life scale. In addition, this study used WHOQOL-BREF as an important reference framework, and perhaps this framework has neglected other elements that contribute to quality of life and thus need further refinement.

## Conclusion

The purpose of this study was to provide a systematic review of quality of life scales and hemophilia-targeting quality of life scales by using textual analysis approach. First, a general description and analysis of quality of life, development of quality of life measurement, and description of hemophilia were presented. Then, referring to the evaluation dimensions of the WHOQOL-BREF as a blueprint, the article came up for a discussion of the development of a universal quality of life scale and a hemophilia-targeting quality of life scale.

The article found that universal quality of life scales mostly fit into the WHOQOL-BREF dimensional framework, with a focus on psychological and social factors. The development of the Hemophilia Quality of Life Scale paid more attention to patient pain and advances in medical technology, but like most quality of life scales, it was deficient in consideration of those indicators such as energy, sleep, sexual life, and recreational participation.

The current hemophilia quality of life scale is dominated by the HR-QoL, and most of the other scales have been developed deeming the HR-QoL a master version. It is worth noting that, unlike the universal quality of life scale, the hemophilia quality of life scale has a distinction between adults and children, which is related to the characteristics of hemophilia onset and treatment. Currently, regional hemophilia QoL scales have been developed in Canada, Europe, and the United States. However, the popularization of existing hemophilia QoL scales is not proportioned. For example, in some developing countries like China, the development of hemophilia-targeting QoL scales is insufficient. Therefore, to develop various hemophilia QoL scales for regions is of significance.

## Author contributions

XW: Data curation, Investigation, Project administration, Writing – original draft. ZL: Conceptualization, Data curation, Formal analysis, Investigation, Methodology, Writing – original draft. LL: Conceptualization, Data curation, Formal analysis, Funding acquisition, Investigation, Methodology, Project administration, Resources, Writing – original draft, Writing – review & editing.
